# Recent Academic Research on Clinically Relevant Digital Measures: Systematic Review

**DOI:** 10.2196/29875

**Published:** 2021-09-15

**Authors:** Md Mobashir Hasan Shandhi, Jennifer C Goldsack, Kyle Ryan, Alexandra Bennion, Aditya V Kotla, Alina Feng, Yihang Jiang, Will Ke Wang, Tina Hurst, John Patena, Simona Carini, Jeanne Chung, Jessilyn Dunn

**Affiliations:** 1 Department of Biomedical Engineering Duke University Durham, NC United States; 2 Digital Medicine Society Boston, MA United States; 3 Big Ideas Lab Department of Biomedical Engineering Duke University Durham, NC United States; 4 Activinsights Ltd Cambridgeshire United Kingdom; 5 Brown-Lifespan Center for Digital Health Brown University Providence, RI United States; 6 Division of General Internal Medicine University of California San Francisco, CA United States; 7 Department of Biostatistics & Bioinformatics Duke University Durham, NC United States

**Keywords:** digital clinical measures, academic research, funding, biosensor, digital measures, digital health, health outcomes

## Abstract

**Background:**

Digital clinical measures collected via various digital sensing technologies such as smartphones, smartwatches, wearables, ingestibles, and implantables are increasingly used by individuals and clinicians to capture health outcomes or behavioral and physiological characteristics of individuals. Although academia is taking an active role in evaluating digital sensing products, academic contributions to advancing the safe, effective, ethical, and equitable use of digital clinical measures are poorly characterized.

**Objective:**

We performed a systematic review to characterize the nature of academic research on digital clinical measures and to compare and contrast the types of sensors used and the sources of funding support for specific subareas of this research.

**Methods:**

We conducted a PubMed search using a range of search terms to retrieve peer-reviewed articles reporting US-led academic research on digital clinical measures between January 2019 and February 2021. We screened each publication against specific inclusion and exclusion criteria. We then identified and categorized research studies based on the types of academic research, sensors used, and funding sources. Finally, we compared and contrasted the funding support for these specific subareas of research and sensor types.

**Results:**

The search retrieved 4240 articles of interest. Following the screening, 295 articles remained for data extraction and categorization. The top five research subareas included operations research (research analysis; n=225, 76%), analytical validation (n=173, 59%), usability and utility (data visualization; n=123, 42%), verification (n=93, 32%), and clinical validation (n=83, 28%). The three most underrepresented areas of research into digital clinical measures were ethics (n=0, 0%), security (n=1, 0.5%), and data rights and governance (n=1, 0.5%). Movement and activity trackers were the most commonly studied sensor type, and physiological (mechanical) sensors were the least frequently studied. We found that government agencies are providing the most funding for research on digital clinical measures (n=192, 65%), followed by independent foundations (n=109, 37%) and industries (n=56, 19%), with the remaining 12% (n=36) of these studies completely unfunded.

**Conclusions:**

Specific subareas of academic research related to digital clinical measures are not keeping pace with the rapid expansion and adoption of digital sensing products. An integrated and coordinated effort is required across academia, academic partners, and academic funders to establish the field of digital clinical measures as an evidence-based field worthy of our trust.

## Introduction

Digital clinical measures are health outcomes or physiological characteristics of an individual’s health, wellness, or condition that are collected digitally with a sensor [1]. Digital sensing products enable rapid assessment of health outcomes and support remote and longitudinal monitoring of patients with chronic diseases under daily living conditions [2-5]. During the COVID-19 pandemic, the utility of digital sensor technologies in clinical research [6], clinical care [7], and public health [8,9] have become even more apparent.

In recent years, digital clinical measures have drawn substantial interest from industry, government agencies, academia, and nonprofit institutions, as digital sensing tools, including consumer products and medical devices, are becoming increasingly popular. Consumer products such as smartwatches and smartphones have become part of daily life for many Americans. These have emerged as popular and multipurpose real-time physiological monitoring products capable of measuring sleep and stress in addition to the more traditional actigraphy and heart rate monitoring. In 2020, 26% of Americans owned a smartwatch [10] and 72% of Americans owned a smartphone [11], with annual sales of over US $70 billion [12]. Apart from consumer products, digital sensing products have demonstrated their efficacy as medical devices, both in clinical and remote home monitoring settings to continuously assess vital signs [13], pulmonary congestion in patients with heart failure [14,15], blood and interstitial glucose in patients with diabetes [3], and more.

To support the development and assessment of digital consumer products and medical devices, the volume of academic research has increased across the total product life cycle of digital clinical measures [16]. However, academic contributions to advancing the safe, effective, ethical, and equitable use of digital clinical measures are poorly characterized and, we hypothesize, underfunded. Trust in digital clinical measures is limited, and engaging the academic community is essential to ensure that the field evolves to be worthy of public trust.

For these reasons, a multi-stakeholder group of experts collaborating on The Playbook [1], a precompetitive collaborative of experts in digital health convened by the Digital Medicine Society (DiMe), set out to investigate the nature of academic research related to digital clinical measures. DiMe is a nonprofit professional society dedicated to advancing digital medicine to optimize health [17]. In this systematic review, we explore the representation of subtypes of academic research on digital clinical measures and compare and contrast the funding support for these subareas of research. This systematic review aims to describe the nature of academic research into digital clinical measures, identify areas of focus and gaps, and explore how and whether funding plays a role. With these findings, we hope to establish an integrated and coordinated effort across academia, academic partners, and academic funders to ensure that the expertise within the field is harnessed to ensure that the rapidly expanding domain of digital clinical measures is established as an evidence-based field worthy of our trust.

## Methods

### Screening

We conducted a systematic search of peer-reviewed literature indexed in PubMed and published between January 1, 2019, and February 24, 2021. For the purposes of this review, we did not restrict the scope of our search to any single digital clinical measure or area of academic research. A multi-stakeholder team of clinical, academic, technical, and operational experts developed the search terms (Multimedia Appendix 1), inclusion criteria (Textbox 1), and selection of data to be extracted from the final publications (Table 1). A biomedical librarian supported the development of the search terms.

**Inclusion criteria adopted to enable the identification of clearly defined academic research related to digital clinical measures.**

**Research lead**

US-led research (ie, ≥50% US-based authors)

**Academic research**

Academic research with at least one US-based academic researcher (industry-only research articles were excluded)

**Article types**

Peer-reviewed journals and full-length conference articles (systematic reviews, meta-analyses, editorials, opinion pieces, case reports, and case studies were excluded)

**Sensing modality**

All portable biometric monitoring technologies (BioMeTs) that rely upon a biometric sensor, such as microphones and accelerometers [16]. BioMeTs are connected digital medicine products that process data captured by mobile sensors using algorithms to generate measures of behavioral or physiological function. (Note: smartphone apps are excluded if they do not rely upon a biometric sensor.)

**Publication date**

Papers published between January 1, 2019, and February 24, 2021

**Table 1:** Data fields extracted from identified academic research.

Field	Definition	Allowed values
Title	N/A^a^	Free text
Authors	Last name, first name	Free text
Author affiliation	N/A	Free text
Journal	Name	Free text
Year	N/A	2019, 2020, 2021
DOI	Digital object identifier: a unique alphanumeric string used to identify content and provide a persistent link to the manuscript’s online location.	Free text
Nature of academic research	Academic research measured here by the publication of peer-reviewed journals and full-length conference articles by study teams that include researchers from either a university or academic institute and society or nonprofit foundation.	Verification, analytical validation, measure identification, clinical validation, security, ethics, data rights and governance, usability and utility (human factors/behavioral economics), standards, usability and utility (data visualization), economic feasibility, operations (care), operations (research design), operations (research analysis), and operations (data)
Digital clinical measure	Health outcomes or physiological characteristics of an individual’s health, wellness, or condition that are collected digitally with a sensor [1]	Biochemical, movement and activity, physiological (electrical, mechanical, optics and imaging)
Funding sources	Funding information	Government, industry, independent foundation, and unfunded

^a^N/A: not applicable.

Following the PubMed search, we conducted a multistep review process to screen articles for inclusion following the PRISMA (Preferred Reporting Items for Systematic Reviews and Meta-Analyses) guidelines [18]. First, we used natural language processing (ie, a custom built Python script; provided in Multimedia Appendix 2 and available on the digital biomarker discovery pipeline [19]) to select papers based on the “Research Lead” and “Academic Research” criteria (Textbox 1). We also excluded articles with “Review” in the title in this step. Second, two of our three trained analysts (authors MMHS, KR, and AB) independently reviewed each publication title against the inclusion criteria. Third, each remaining abstract was reviewed by two of the three analysts (MMHS, KR, and AB) to determine whether the article met our inclusion criteria (Textbox 1). When there was disagreement between two reviewers during either the title or abstract review phase, the decision whether to advance a publication was resolved by the third analyst. Finally, two of three analysts (MMHS, KR, and AB) reviewed the full text of each of the publications that passed the abstract screening stage, with the involvement of a third analyst to settle interrater disagreements (approximately 15% of papers reviewed), to establish the final list of publications for inclusion. The list of articles excluded from the full-text screening process is given in Multimedia Appendix 3.

### Data Extraction and Categorization

Following the screening phase, seven analysts (authors MMHS, KR, AB, AVK, AF, YJ, and WKW) extracted data from the articles included in the data extraction phase and categorized each publication as described in Table 1. The articles were categorized according to the following three criteria: nature of academic research, category of digital clinical measures, and source of funding.

The categories to subgroup the “nature of academic research” included verification, analytical validation, measure identification, clinical validation, security, ethics, data rights and governance, usability and utility (human factors and behavioral economics), standards, usability and utility (data visualization), economic feasibility, operations (care), operations (research design), operations (research analysis), and operations (data).

The categories to subgroup “digital clinical measures” included biochemical, movement and activity, and physiological (electrical, mechanical, and optics and imaging).

“Funding sources” were subgrouped by government, industry, independent foundation, and unfunded. Articles with missing funding information were categorized as unfunded. The details of these categories are given in Table 2.

**Table 2:** Categories for data extraction.

Category			Definitions		Reference
**Nature of academic research**					
	Verification	Evaluates and demonstrates the performance of a sensor technology within a BioMeT^a^, and the sample-level data it generates, against a prespecified set of criteria		[16]	
	Analytical validation	Evaluates the performance of the algorithm, and the ability of this component of the BioMeT to measure, detect, or predict physiological or behavioral metrics		[16]	
	Clinical validation	Evaluates whether a BioMeT acceptably identifies, measures, or predicts a meaningful clinical, biological, physical, functional state, or experience in the stated context of use (which includes a specified population)		[16]	
	Measure identification	Research studies to identify key variables from the information extracted from digital sensors, to support decision-making		[20]	
	Security	Research studies to assess the risks associated with digital clinical measures and taking necessary measures for information security		[21]	
	Data rights and governance	Research studies to assess the data access, privacy, and sharing (following the FAIR^b^ guiding principle)		[22]	
	Ethics	Research studies to ensure equity and justice during every step of the development and deployment of digital clinical measures (eg, reduce health disparities or racial injustice)		[23]	
	Usability and utility (human factors/behavioral economics)	Research studies to investigate human factors associated with digital clinical measures (eg, how usable, useful, or unobtrusive a digital clinical measure can be for an end user). It involves surveys from the participants on user experience.		[24]	
	Standards	Involves standardization of the data extracted from digital clinical measures for interoperability		[25]	
	Usability and utility (data visualization)	Involves data visualization/result presentation for all end uses		[24]	
	Economic feasibility	Research studies to investigate economic feasibility of a digital clinical measure		[26]	
	Operations (care)	Involves clinicians and economists to design clinical workflow and corresponding evaluation that is typically done for a clinical trial		[27]	
	Operations (research design)	Involves clinicians and biostatisticians to design a research study and execution plan, which is typically done for a clinical trial via power analysis and statistical analysis plan		[28]	
	Operations (research analysis)	Involves analyzing data from digital clinical measures (eg, data analyst or data scientists)		[29]	
	Operations (data)	Involves monitoring data and metadata from digital clinical measures (eg, bioinformatics)		[30]	
**Digital clinical measures**					
	Biochemical	Senses biochemicals (eg, sweat sensor or continuous glucose monitors)		[31]	
	Movement and activity	Tracks movement and activity (eg, step count or actigraph)		[31]	
	Physiological (electrical)	Senses electrical signals related to physiological phenomena (eg, electrocardiography, electroencephalography, electromyography, bioimpedance, electrodermal activity, or electroooculography)		[32-34]	
	Physiological (mechanical)	Senses mechanical signals related to physiological phenomena (eg, phonocardiography, speech, lung sounds, joint acoustic emission, seismocardiography, or ballistocardiography)		[35,36]	
	Physiological (optics and imaging)	Senses optical signals related to physiological phenomena (eg, photoplethysmography, camera for blood volume pulse, or bioradar)		[37]	
**Funding sources**					
	Government	US Government funding agencies		[38]	
	Industry	Pharma, tech, and medical device industry		[38]	
	Independent foundation	Universities, private nonprofits, societies, and independent associations		[38]	
	Unfunded	Investigator initiated with no funding sources explicitly stated			

^a^BioMeT: biometric monitoring technology.

^b^FAIR: Findable, Accessible, Interoperable, and Reusable.

For the data extraction process, each publication was reviewed by at least three of the seven analysts (MMHS, KR, AB, AVK, AF, WKW, and YJ). Each publication was assigned to one or more categories of a particular criterion as a result of two or more votes for a particular category for each publication. This method of subgrouping was used to reduce the impact of individual analyst subjectivity at this stage. Following the initial categorization, articles falling into the government funding subgroup were further categorized by US government agency (ie, National Institutes of Health [NIH], National Science Foundation [NSF], Department of Defense [DOD], Veteran Affairs [VA], National Aeronautics and Space Administration [NASA], Department of Energy [DOE], and “Other”). The “Other” category constitutes government funding sources that were listed for just one article in our pool. Articles with NIH funding were further subgrouped by NIH institutes and centers [39]. Data was standardized after extraction by the five analysts (MMHS, KR, AVK, AF, and AB), and the details of this process are presented in Multimedia Appendix 4.

Following the data extraction process, we performed Pearson chi-square tests with one categorical variable to determine whether the representation of academic research studies varies significantly within the following categories: academic research, digital sensors, and funding sources. We assumed equal representation for all categories as the null hypothesis. In this work, we considered *P* values less than .05 to be statistically significant. The statistical tests and data visualization were performed using Python 3.8.5 (Python Software Foundation) on the Spyder-integrated development environment 4.1.5.

## Results

### Screening

**Figure 1:**
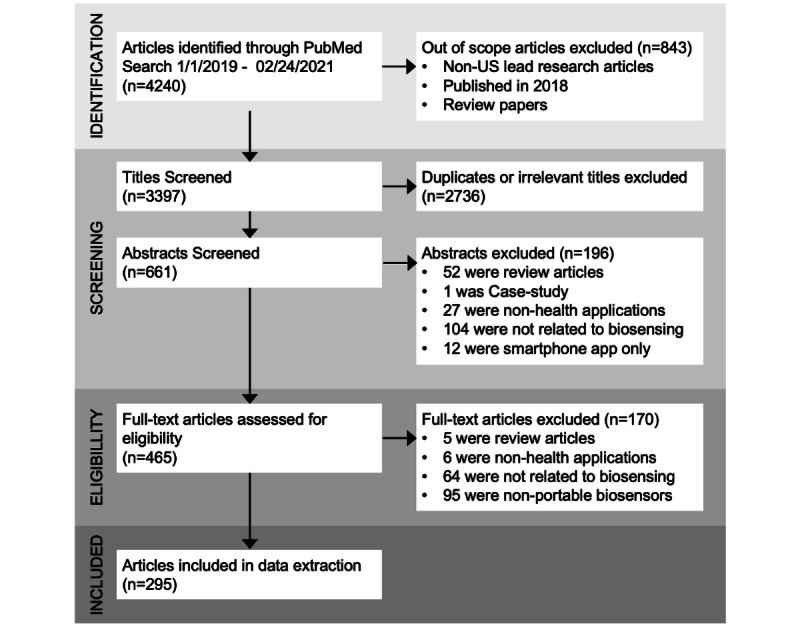
Article screening process and diagram following the PRISMA (Preferred Reporting Items for Systematic Reviews and Meta-Analyses) review methodology.

Our initial search on PubMed retrieved 4240 articles (Figure 1). With our custom built Python script, we excluded 843 articles from this initial list based on research lead, publication year, and publication type. Of the 3397 identified articles for subsequent screening, we excluded over 75% (n=2736) after title screening and a further 30% (n=196) after abstract screening, based on our inclusion criteria. The majority of the excluded articles were not related to biosensing, were review articles, or explored nonhealth applications. Following the abstract screening, a total of 465 articles were included in the full-text review, during which we further excluded 170 (37%) articles on the basis of our inclusion criteria. At this stage, articles were mainly excluded because the sensors being studied were nonportable biosensors or because they covered topics unrelated to biosensing; Multimedia Appendix 3 lists the articles excluded in this phase. Data for further analysis was extracted from the remaining 295 articles.

### Data Categorization

**Figure 2:**
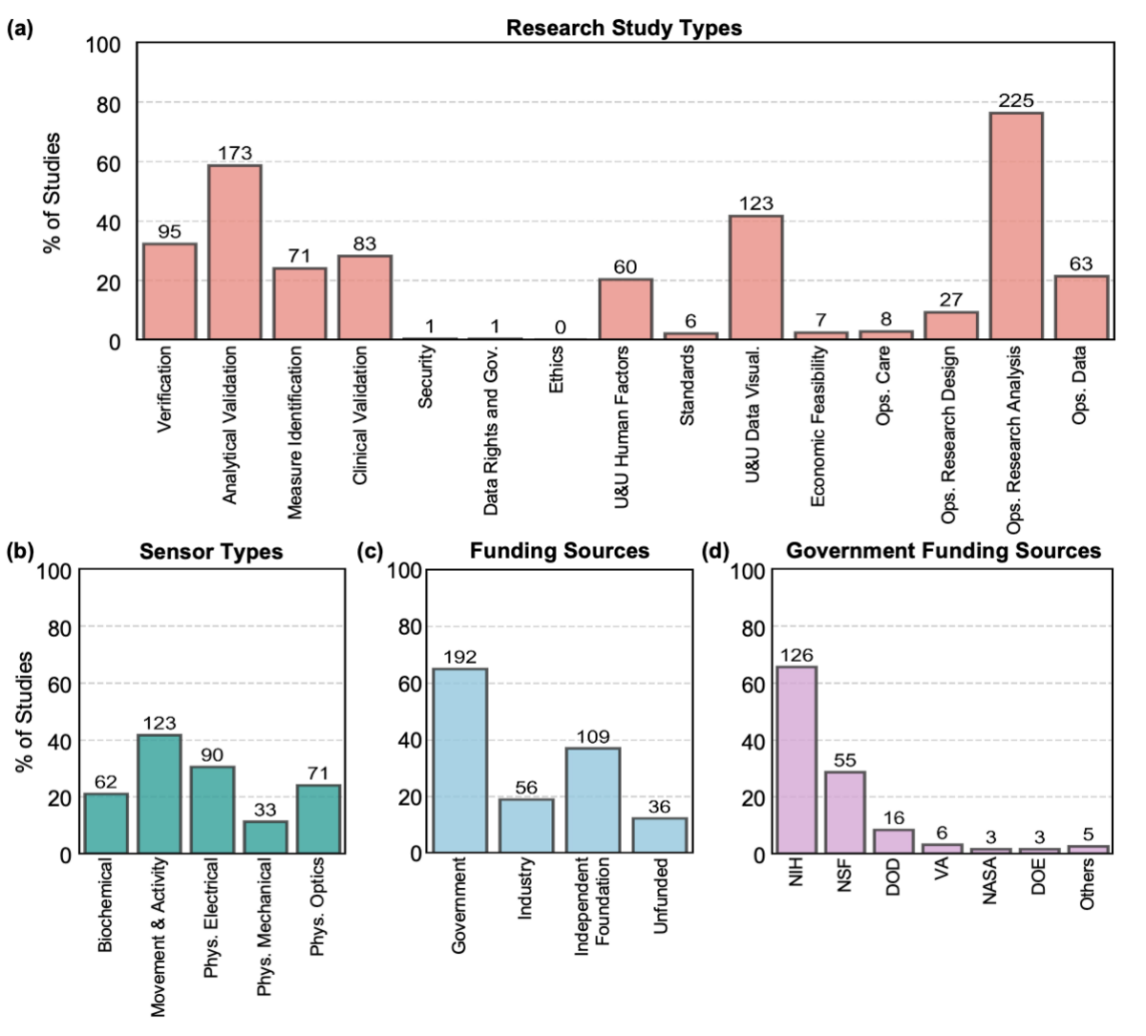
Distribution of articles across (a) research study types, (b) different sensing modalities, (c) different funding sources, and (d) different government funding agencies. The bars are showing the percentage of studies, with 100% equivalent to 295 papers included in the data extraction process for (a-c) and 100% equivalent to 192 papers with government funding for (d). The text on top of the bars in all the plots showing the actual number of articles per category. Others in (d): state governments, National Institute of Justice, US Department of Agriculture, National Institute of Food and Agriculture. One article can be grouped into multiple categories for (a-d). DOD: Department of Defense; DOE: Department of Energy; Gov: governance; NASA: National Aeronautics and Space Administration; NIH: National Institutes of Health; NSF: National Science Foundation; Ops: operations; Phys: physiological; U&U: usability and utility; Visual: visualization; VA: Veteran Affairs.

The 295 articles used for analysis were categorized by research study type, sensor type, and funding source, including broad US government funding sources and specific NIH funding sources (Figure 2). The list of the 295 articles included in the data extraction process with their corresponding categories is located in Multimedia Appendix 4. We observed statistically significant differences (Pearson one-sample chi-square test *P*<.001), indicating unequal distribution of research studies across subcategories for all three overarching categories: academic research, digital sensors, and funding sources. Nearly 76% (n=225) of the studies evaluated were conducted in operations research analysis (Figure 2a). Analytical validation (n=173, 59%), usability and utilities (data visualization; n=123, 42%), verification (n=93, 32%), and clinical validation (n=83, 28%) were other commonly represented study types. On the contrary, ethics (n=0), security (n=1), and data rights and governance (n=1) were uncommon study types. Research on standards (n=6), economic feasibility (n=7), and operations care (n=8) were also uncommon in this pool of articles.

Categorization by sensor types (Figure 2b) revealed that movement and activity were the most commonly studied sensors in the article pool (n=123, 42%), followed by physiological (electrical) sensors (n=90, 31%), physiological (optics and imaging) sensors (n=71, 24%), biochemical sensors (n=62, 21%), and physiological (mechanical) sensors (n=33, 11%). For those studies evaluating movement and activity sensors, actigraphy and activity monitors with wearable accelerometers were the most commonly studied sensors.

Studies categorized by funding source (Figure 2c) indicated that government agencies are funding the majority (n=192, 65%) of academic research on digital clinical measures, followed by independent foundations (n=109, 37%) and industry (n=56, 19%). Interestingly, more than 1 in 10 digital clinical measures studies (n=36, 12%) was unfunded. Of these unfunded studies, 22 articles explicitly stated that the research team did not receive any external funding, and 14 did not include a statement on funding.

**Figure 3:**
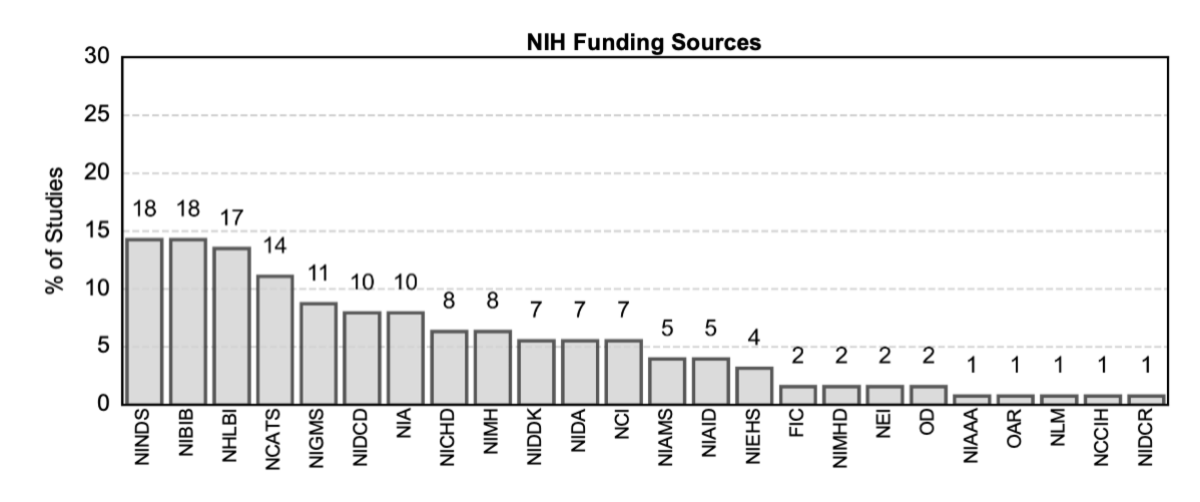
Distribution of articles across different NIH institutes and centers. The bars are showing the percentage of studies, with 100% equivalent to 126 papers with NIH funding. The text on top of the bars showing the actual number of articles per category. One article can be grouped into multiple categories. FIC: Fogarty International Center; NBIB: National Institute of Biomedical Imaging and Bioengineering; NCATS: National Center for Advancing Translational Sciences; NCCIH: National Center for Complementary and Integrative Health; NEI: National Eye Institute; NHLBI: National Heart, Lung, and Blood Institute; NIA: National Institute on Aging; NIAAA: National Institute on Alcohol Abuse and Alcoholism; NICHD: National Institute of Child Health and Human Development; NIDA: National Institute on Drug Abuse; NIDCD: National Institute on Deafness and Other Communication Disorders; NIDCR: National Institute of Dental and Craniofacial Research; NIDDK: National Institute of Diabetes and Digestive and Kidney Diseases; NIEHS: National Institute of Environmental Health Sciences; NIGMS: National Institute of General Medical Sciences; NIH: National Institutes of Health; NIMH: National Institute of Mental Health; NIMHD: National Institute on Minority Health and Health Disparities; NINDS: National Institute of Neurological Disorders and Stroke; NLM: National Library of Medicine; OAR: Office of AIDS Research; OD: Office of Dietary Supplements.

For studies receiving government funding, the NIH was the most frequent contributor in terms of the number of articles funded—66% (n=126) of the studies with government funding were funded by the NIH (Figure 2d). The NSF was the second most frequent government funder of research on digital clinical measures (n=55, 29%), followed by DOD (n=16, 8%), VA (n=6, 3%), NASA (n=3, 2%), DOE (n=3, 2%), and others (the 5 remaining studies were funded by state governments, National Institute of Justice, US Department of Agriculture, or National Institute of Food and Agriculture). Of the 27 institutes and centers at the NIH [39], 24 institutes funded studies on digital clinical measures (Figure 3), indicating widespread interest and applications in this field. The majority of studies were funded by the National Institute of Neurological Disorders and Stroke (n=18, 14% of the studies with NIH funding); the National Institute of Biomedical Imaging and Bioengineering (n=18, 14%); the National Heart, Lung, and Blood Institute (n=17, 13%); and the National Center for Advancing Translational Sciences (n=14, 11%).

Of the articles that reported receiving funding from independent foundations (n=109), 75 (69%) studies received funding from institutional funds at universities, 46 (42%) studies received funding from private nonprofits (eg, Bill and Melinda Gates Foundation or Chan Zuckerberg Initiative), and 6 (6%) received funding from societies and associations (eg, American Heart Association).

**Figure 4:**
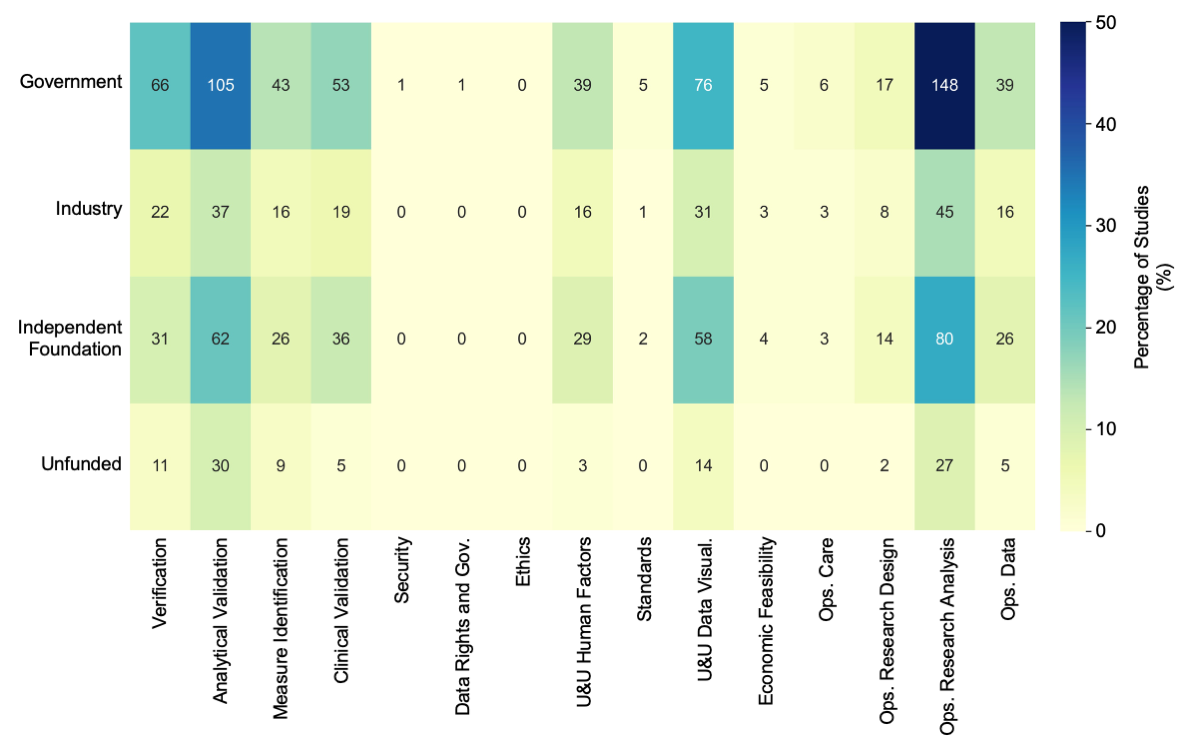
Distribution of funding sources across different research study types, with the heat map color showing the percentage of studies (100% equivalent to 295 papers included in the data extraction process) and the text in each cell showing the actual number of articles per category. One article can be grouped into multiple categories. Gov: governance; Ops: operations; U&U: usability and utility; Visual: visualization.

To understand whether specific funding types may be driving specific sectors of digital clinical measures research, and where a lack of funding may be contributing to low research output, we explored the distribution of funding across different research study types (Figure 4). Subdividing the research topics by funding type, we found that the proportion of research support from each of the four funding categories was fairly consistent across research areas. The most frequent research and funding combination out of the 295 articles was operations research analysis supported by government funding (n=148, 50%). The second most common combination was analytical validation studies supported by government funding (n=105, 36% of overall studies). Operations research analysis and analytical validation also represent the first- and second-largest sectors of overall digital clinical measures research, respectively (Figure 2a). Interestingly, the third most frequent research and funding combination was not another research category but rather an operations research analysis funded by foundations (n=80, 27% of overall studies), indicating the large overall footprint that operations research analysis occupies in the academic digital clinical measures research space. By contrast, even given the large proportion of government funding, research categorized as analytical validation was also the most likely to be unfunded, with 30 out of the 173 (17%) studies reported as unfunded.

**Figure 5:**
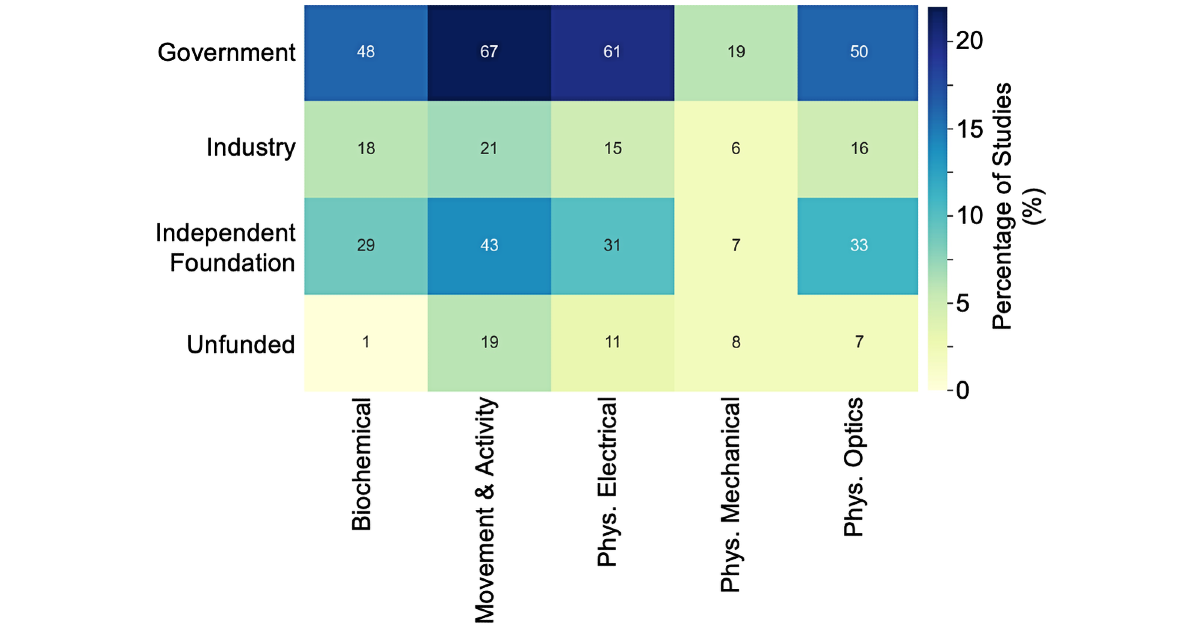
Distribution of funding sources across different digital sensor types, with the heat map color showing the percentage of studies (100% equivalent to 295 papers included in the data extraction process) and the text in each cell showing the actual number of articles per category. One article can be grouped into multiple categories. Phys: physiological.

Similar to the previous analysis subdividing research topics by funding type, we sought to understand whether the volume of literature surrounding particular types of digital sensors is related to funding. Therefore, we subdivided the articles in the different digital sensor categories from Figure 2b by funding type. We found that the most frequent digital sensor and funding combination was movement and activity sensors funded by government agencies (n=67, 23% of overall studies; Figure 5). Government funding also supported the majority of research into physiological (electrical) sensors (n=61, 21%), physiological (optics and imaging) sensors (n=50, 17%), and biochemical sensors (n=48, 16%). By contrast, even given the large proportion of government funding, digital sensors categorized as movement and activity were the second most likely to be unfunded, with 15% (n=19) of the 123 studies on movement and activity sensors being unfunded. Biochemical sensors were the least likely to be studied without funding (n=1, 1% of studies that used biochemical sensors), whereas studies into physiological (mechanical) sensors were most the commonly unfunded, with 8 of 33 (24%) studies reported as unfunded.

## Discussion

### Principal Findings

In this systematic review, we describe the nature of academic research related to digital clinical measures and the distribution of funding across different types of academic research and sensing modalities.

Verification, analytical validation, and clinical validation studies [16] are, together, the most frequently published study types in this review. As verification, analytical validation, and clinical validation is foundational to establishing whether a digital clinical measure is fit-for-purpose [16], these findings indicate that academic research supporting the development and evaluation of digital clinical measures is appropriate for a nascent field. However, the paucity of published studies examining the security, data rights and governance, ethics, standards, and economic feasibility of digital sensing products are alarming given the rapid growth and adoption of digital clinical measures [40]. The risks of harm to individuals from unauthorized access to data arising from inadequate security, misuse of data due to poor data rights and governance, and inequities arising from the development and deployment of digital clinical measures without sufficient consideration of the ethical implications are substantial [21,23,41,42]. It is imperative that academic investigators skilled in these areas are motivated and funded to pursue a systematic evaluation of the current state of affairs and to propose best practices to ensure that digital clinical measures fulfill their promise without causing harm to individuals or populations.

Research studies examining the usability and utility of digital sensing products are relatively common compared to publications reporting research into security, data rights and governance, and economic feasibility, which ought to trend together [24]. This is not to say that usability and utility of digital clinical measures is overstudied, but rather suggests that research into these other characteristics of digital sensing products is lagging. Similarly, the number of publications reporting measure identification is relatively low compared to research into the development and deployment of these same measures. This may be cause for concern if we cannot be certain that digital clinical measures being developed have already been determined to be clinically relevant and grounded in aspects of health that patients and clinicians care most about [20]. As we strive to increase the patient focus and efficiency of health care, it is critical that we are separating signals from noise and not advancing digital clinical measures that offer little value to individual patients and the health care system.

Research into the operational aspects of deploying digital clinical measures is the largest single study type identified by our review. Although digital clinical measures cannot add value unless they are successfully operationalized during routine clinical care and in clinical trials, focusing academic research on deployment without first ensuring that the digital clinical measures are fit-for-purpose and trustworthy leaves the entire field of digital health at risk of collecting vast swaths of data that, at best, are of no value and, at worst, could cause harm. During the rapid acceleration of digital clinical measurements, research into the selection and development of high-quality measures and tools must be a primary focus of academic research in this new field.

Research related to movement and activity sensors are most common when we parse the article pool by sensor type. This finding is consistent with other literature where digital measures of activity have been found to be most commonly used to answer clinical questions [43]. Movement and activity sensors also inform the majority of digital end points used by the industry in medical product development [40]. Physiological (electrical), physiological (optical and imaging), and biochemical sensors are well represented in this review, which is consistent with the recent growth in the use of portable electrocardiograms, photoplethysmography, and continuous glucose monitoring, respectively.

Our review indicates that government agencies and independent foundations are funding most of the academic research studies related to digital clinical measures. Industry funding was relatively low, and this is likely due to our definition of academic studies that excludes studies that only have industry-affiliated authors without academic research partnerships. Of the government agencies, the NIH is funding most of the academic research studies, which is consistent with previous research examining funding of US biomedical research [38]. The distribution of funding across different NIH institutes and centers demonstrates that certain therapeutic areas might be getting more funds compared to others. However, we have not extracted information on funding distribution across different therapeutic areas, as it was out of scope for this systematic review. Future work should explore which therapeutic areas are more likely to receive funding and which areas are least funded. Of the independent foundation–funded research studies, institutional funds at universities are funding the majority on digital clinical measures as compared with private nonprofits and public charities, which is also consistent with the literature [38].

After operational research, analytical validation is the most common government-funded study type in digital clinical measurement. This is critically important as analytical validation includes examination of algorithmic bias [16], which must be an area of focus given research findings that digital sensing products may not perform equally well across different skin tones, among other factors [44,45]. However, although the total number of government-funded analytical validation publications is high, analytical validation studies are also the most likely to be unfunded (n=30, 17%), suggesting that academic researchers are pursuing analytical validation studies even when funding may not exist. This work is to be applauded but is not sustainable. Additional funding for analytical validation must be made available to ensure that digital clinical measures are developed equitably.

Although movement and activity sensors are the most used sensors in academic research, these sensors are still the second most likely to be unfunded (n=19, 15%), suggesting that academic researchers are pursuing research into movement and activity sensors even when funding may not exist. This is again praiseworthy but not sustainable, considering the rapid adoption of these sensors in our daily life [10,11] and clinical studies [43]. Sufficient funding is required to ensure the development and deployment of these movement and activity sensors reliably and equitably.

Our review has several limitations. First, we have focused only on academic research led by US-based academic researchers. Future research should expand beyond the United States to examine trends in academic research into digital clinical measures globally. Second, we searched only one database (PubMed) to retrieve articles for this review. PubMed only indexes research related to life sciences and biomedicine [46]. As digital medicine is a highly interdisciplinary field, many relevant studies may not have been captured in our review. For example, sensor verification studies may be published in traditional engineering journals that are not indexed by PubMed. Future studies will be enhanced by the use of multiple databases across disciplines. In addition, as an emerging interdisciplinary field, we must strive to reference the complete corpus of relevant literature, not only those publications familiar to us in our individual disciplines. Finally, the subjective nature of the review and data extraction process may hinder repeatability, and we attempted to mitigate this risk using innovative methods such as using a majority voting system and using natural language processing to automate the initial screening phase.

This review reports the current state of academic research on the rapidly expanding and highly promising field of digital clinical measures. Substantial work is being done in areas such as validation and operations, with a paucity of research in other areas like security and ethics. Future studies should investigate why critical research into the safe, effective, ethical, and equitable advancement of digital clinical measures is largely absent from the published literature. Both academic researchers and funding agencies should focus on the subareas of academic research on digital clinical measures that are underrepresented and relatively underfunded to ensure that funding priorities adequately reflect the evidentiary needs of the field.

### Conclusion

Academic research related to digital clinical measures is not keeping pace with the rapid expansion and adoption of digital sensing products. Although substantial foundational research validating the performance of digital clinical measures is being conducted, academic studies of security, data rights and governance, economic feasibility, ethics, and standards necessary to advance the field are lagging. These areas must be bolstered to minimize the growing chasm between the promised benefits of digital clinical measures and their potential risks. As expected, research funding appears to be associated with increased research publications. An integrated and coordinated effort is required across academia, academic partners, and academic funders to establish the field of digital clinical measures as an evidence-based field worthy of our trust.

## Appendix

Multimedia Appendix 1 PubMed search terms.

Multimedia Appendix 2 Python code.

Multimedia Appendix 3 List of articles excluded after full-text review.

Multimedia Appendix 4 List of included papers with categories.

## Abbreviations

DiMe: Digital Medicine Society
DOD: Department of Defence
DOE: Department of Energy
NASA: National Aeronautics and Space Administration
NIH: National Institutes of Health
NSF: National Science Foundation
PRISMA: Preferred Reporting Items for Systematic Reviews and Meta-Analyses
VA: Veteran Affairs
