# Supplementary material for: Recent Academic Research on Clinically Relevant Digital Measures: Systematic Review
Source: J Med Internet Res. 2021 Sep 15;23(9):e29875. doi: 10.2196/29875 (PMC8482196; doi:10.2196/29875)
Supplement: Multimedia Appendix 1 [file jmir_v23i9e29875_app1.docx]

**Supplementary Table 1: PubMed search terms**

| **Layer** | **Search Terms** | **Results Returned (search date February 24, 2021)** |
| --- | --- | --- |
| #1 | "instrumentation"[Subheading] OR "Transducers"[Mesh] OR “transducer*"[tiab] OR “instrument*”[tiab] OR “device*”[tiab] OR “equipment*”[tiab] OR "Wireless Technology"[Mesh] OR "remote technolog*"[tiab] OR Wireless[tiab] OR wear[tiab] OR worn[tiab] OR wearable[tiab] OR wearing[tiab] OR "Signal Processing, Computer-Assisted"[Mesh] OR remote[tiab] OR ((“mobile”[tiab] OR “wearable” [tiab]) AND (“data” [tiab] OR “metadata”[tiab])) OR "mobile tech*"[tiab] OR "mobile device*"[tiab] OR “mobile application*”[tiab] “mobile apps”[tiab]OR "wearable technolog*"[tiab] OR "wearable device*"[tiab] OR "accelerometer*"[tiab] OR "biosensor"[tiab] OR "gyroscope*"[tiab] OR "smart watch*"[tiab] OR "smartwatch*"[tiab] OR "wristband*"[tiab] OR "armband*"[tiab] OR "ring"[tiab] OR "earbud*"[tiab] OR "digital health"[tiab] OR "biometric"[tiab] OR "ResearchKit"[tiab] OR "HealthKit"[tiab] OR "healthpatch"[tiab] OR "biochip"[tiab] OR "holter*"[tiab] OR "mobile health data"[tiab] OR ("sensor*"[tiab] AND ("data"[tiab] OR "wireless"[tiab] OR "wearable"[tiab])) OR "digital technology"[MeSH] OR "digital technolog*"[ tiab] OR "unobtrusive"[tiab] OR "noninvasive"[tiab] OR "contact*"[tiab] OR "non-contact"[tiab] OR "skin"[tiab] OR “adhere*”[tiab] OR “implant*”[tiab] OR “ingest*” [tiab] OR “portable”[tiab] OR “embedded”[tiab] OR movement[tiab] OR IMU[tiab] OR ("chest*"[tiab] AND "band"[tiab]) OR (("ankle"[tiab] OR "ankle joint"[tiab]) AND "band"[tiab]) OR "inertial measurement unit"[tiab] OR "IMU"[tiab] OR (("textiles"[MeSH] OR "textile*"[tiab]) AND "sensor*"[ tiab]) OR “microphone*”[tiab] OR “camera*”[tiab] | 2,470,227 |
| #2 | (“test*”[tiab] OR “measure*”[tiab] OR “detect*”[tiab] OR “monitor*”[tiab] OR “record*”[tiab] OR “diagnos*”[tiab]) AND (“compar*”[tiab] OR “validat*”[tiab] OR “reproduc*”[tiab] OR “equivalent”[tiab] OR “verif*”[tiab] OR “analy*”[tiab] OR “prototyp*”[tiab] OR “assess*”[tiab] OR “predict*”[tiab] OR “connect*”[tiab] OR standards[tiab] OR provenance[tiab] OR semantics[tiab] OR representation[tiab]) AND ("Monitoring, Physiologic"[Mesh] OR "Accelerometry"[Mesh] OR "Physical Examination"[Mesh] OR "Movement"[Mesh] OR "physiology"[subheading] OR "physiopathology"[subheading] OR "Signs and Symptoms"[Mesh] OR accelerometry[tiab] OR actigraphy[tiab] OR movement[tiab] OR gait[tiab] OR “pathology*” [tiab] OR “physiopathology*”[tiab] OR “physiologic*”[tiab] OR “function*”[tiab] OR “activit*”[tiab] OR locomotion[tiab] OR consumption[tiab] OR “exercise*”[tiab] OR health[tiab] OR security[tiab] OR “health econom*”[tiab]) | 4,333,773 |
| #3 | #1 AND #2 | 551,321 |
| #4 | #3 NOT (Editorial[ptyp] OR Comment[ptyp] OR meta-analysis[ptyp] OR systematic review[ptyp] OR Case Reports[ptyp] OR "a case study"[ti] OR ": case study"[ti] OR "case study: ") AND English[lang] | 504,029 |
| #5 | #4 AND ( "2019/01/01"[PDat] : "3000/12/31"[PDat]) | 76,073 |
| #6 | #5 AND (USA[Affiliation] OR US[Affiliation] OR United States of America[Affiliation]) | 13,536 |
| #7 | #6 AND ("edu"[Affiliation] OR "org"[Affiliation]) AND ("college"[Affiliation] OR "university"[Affiliation] OR "institute"[Affiliation] OR "society"[Affiliation]) | 4,240 |

PubMed indexing abbreviations: MeSH= Medical Subject Headings®, MH = MeSH Heading; ptyp = publication type; tiab = restricts query to search in the title or abstract of the articles.
