# Supplementary material for: Recent Academic Research on Clinically Relevant Digital Measures: Systematic Review
Source: J Med Internet Res. 2021 Sep 15;23(9):e29875. doi: 10.2196/29875 (PMC8482196; doi:10.2196/29875)
Supplement: Multimedia Appendix 2 [file jmir_v23i9e29875_app2.docx]

**Multimedia Appendix 2**. Python code.

**Code Availability:**

The python script used for the initial screening phase is provided in the following link.

Link: <https://github.com/DigitalBiomarkerDiscoveryPipeline/systematic-digital-biomarker-literature-search>
